# Minimally invasive posterior locked compression plate osteosynthesis shows excellent results in elderly patients with fragility fractures of the pelvis

**DOI:** 10.1007/s00068-020-01498-8

**Published:** 2020-10-26

**Authors:** Imke U. Schmerwitz, Philipp Jungebluth, Wolfgang Lehmann, Thomas J. Hockertz

**Affiliations:** 1 Department of Orthopedic Surgery, Sports Traumatology and Trauma Surgery, Staedtisches Klinikum Wolfenbuettel, Alter Weg 80, 38302 Wolfenbuettel, Germany; 2 Department of Trauma, Orthopedic and Plastic Surgery, University Medical Center Goettingen, Goettingen, Germany

**Keywords:** Fragility fracture, Sacrum, Insufficiency fracture, Posterior locked compression plate

## Abstract

**Purpose:**

Fragility fractures of the pelvis (FFP) are common in older patients. We evaluated the clinical outcome of using a minimally invasive posterior locked compression plate (MIPLCP) as therapeutic alternative.

**Methods:**

53 Patients with insufficiency fractures of the posterior pelvic ring were treated with MIPLCP when suffering from persistent pain and immobility under conservative treatment.

After initial X-ray, CT-scans of the pelvis were performed. In some cases an MRI was also performed to detect occult fractures. Postoperatively patients underwent conventional X-ray controls. Data were retrospectively analyzed for surgical and radiation time, complication rate, clinical outcome and compared to the literature.

**Results:**

Patients (average age 79.1 years) underwent surgery with operation time of 52.3 min (SD 13.9), intra-operative X-ray time of 9.42 s (SD 9.6), mean dose length product of 70.1 mGycm (SD 57.9) and a mean hospital stay of 21.2 days (SD 7.7).

13% patients (*n* = 7) showed surgery-related complications, such as wound infection, prolonged wound secretion, irritation of the sacral root or clinically inapparent screw malpositioning. 17% (*n* = 9) showed postoperative complications (one patient died due to pneumonia 24 days after surgery, eight patients developed urinal tract infections).

42 patients managed to return to previous living situation. 34 were followed-up after a mean period of 31.5 (6–90) months and pain level at post-hospital examination of 2.4 (VAS) with an IOWA Pelvic Score of 85.6 (55–99).

**Conclusion:**

We showed that MIPLCP osteosynthesis is a safe surgical alternative in patients with FFP 3 and FFP 4. This treatment is another way of maintaining a high level of stability in the osteoporotic pelvic ring with a relatively low complication rate, low radiation and moderate operation time and a good functional outcome.

## Introduction

Osteoporosis related fractures are common in older patients and its prevalence is rising with advancing age [1, 2]. Even though a high number of fractures involve the wrist, hip and spine the number of osteoporotic pelvic fractures including sacral and pubic rami fractures is likewise increasing rapidly [3, 4]. The incidence of pelvic fractures in the German population aged 60 years or older was 22.4 per 10,000 person years. A significant age and sex effect on first fracture risk was confirmed [3].

The number of people affected by osteoporotic fractures is increasing worldwide just as the older population [5]. However, the social and economic burden will unavoidably become more and more relevant. In the United States 7% of all osteoporosis related fractures in people over 50 years of age are pelvic fractures which accounts for 5% of the total economic costs [1].

Studies revealed a substantial one-year mortality of about 8–27% in pelvic fractures with excess mortality being highest in the first four weeks after the occurrence of the fracture [6]. There was also a high number of patients moving from an independent to a dependent living situation, which was similar to data reported for elderly patients with a hip fracture [7].

However it is still not thoroughly investigated which portion of the excess mortality is caused by the fracture itself or caused by associated prior existing comorbidities and poor health status [8].

Low energy trauma such as sliding of a chair or even lack of trauma, may result in an unstable pelvic fracture which can lead to immobilizing pain. The loss of elasticity and strength, hence a reduced bone quality results in so called fragility fractures of the pelvis.

Recent studies on osteoporosis related pelvic fractures demonstrated an increase of incident rates over the last decade [3, 9, 10]. Part of the increase may be due to improved diagnostic methods (CT, MRI) and greater awareness of the fracture pattern, as well as to an increase of life expectancy. Still, the overall impact of pelvic fractures is immense.

The World Health Organization (WHO) defines a fragility fracture as one caused by a trauma that would be insufficient to injure bone if the bone substance were normal.

In some cases particular isolated fractures of the sacrum cannot immediately be detected, but are only seen in the magnetic resonance imaging (MRI scan) [11]. Patients suffer from severe lower back pain and loss of mobility.

In 2013, the Fragility Fractures of the Pelvis classification was introduced for this fracture entity [12]. The classification is based on instability signs and includes treatment recommendations. Nevertheless, there are still controversies regarding when and how these fractures should be treated. In recent years it has become more and more obvious that fragility fractures of the pelvis are a different entity in comparison to traumatic pelvic fractures due to different fracture mechanism, bone quality and degree of instability [13, 14].

In patients with anterior fractures only, conservative treatment with early mobilization and pain relief medication is highly recommended [14, 15]. However, patients with combined posterior and anterior pelvic fractures suffer from severe pain when being mobilized; surgical treatment should be considered to avoid institutionalization. There are a number of operative procedures available, such as percutaneous iliosacral screw fixation, sacroplasty, lumbo-pelvic stabilization, sacral bar fixation with or without additional ventral fixation, transiliac internal fixator and minimally invasive posterior locked compression plate (MIPLCP) [16–29].

In this paper, outcomes of the MIPLCP osteosynthesis as a standalone treatment option in pelvic fractures, which was first applied for polytraumatized patients [26], was applied to elderly patients with osteoporotic bone stock.

The purpose of our study was to determine whether the MIPLCP osteosynthesis shows equal results regarding complication rate and outcome as ilio-sacral-screw osteosynthesis and whether operation time and radiation time is lower.

## Patients and methods

### Inclusion criteria

After ethical committee approval, a retrospective data analysis of patients with fragility fractures of the pelvis was performed. Inclusion criteria consisted of age above 60 years with low energy trauma. Surgical treatment was indicated in fractures we considered as being unstable such as FFP 3 and FFP 4 fractures and in some cases of FFP 2 fractures with persistent pain under conservative treatment. Additional surgical treatment of the pelvis let to exclusion of the study cohort.

### Diagnostics and treatment

**Fig. 1:**
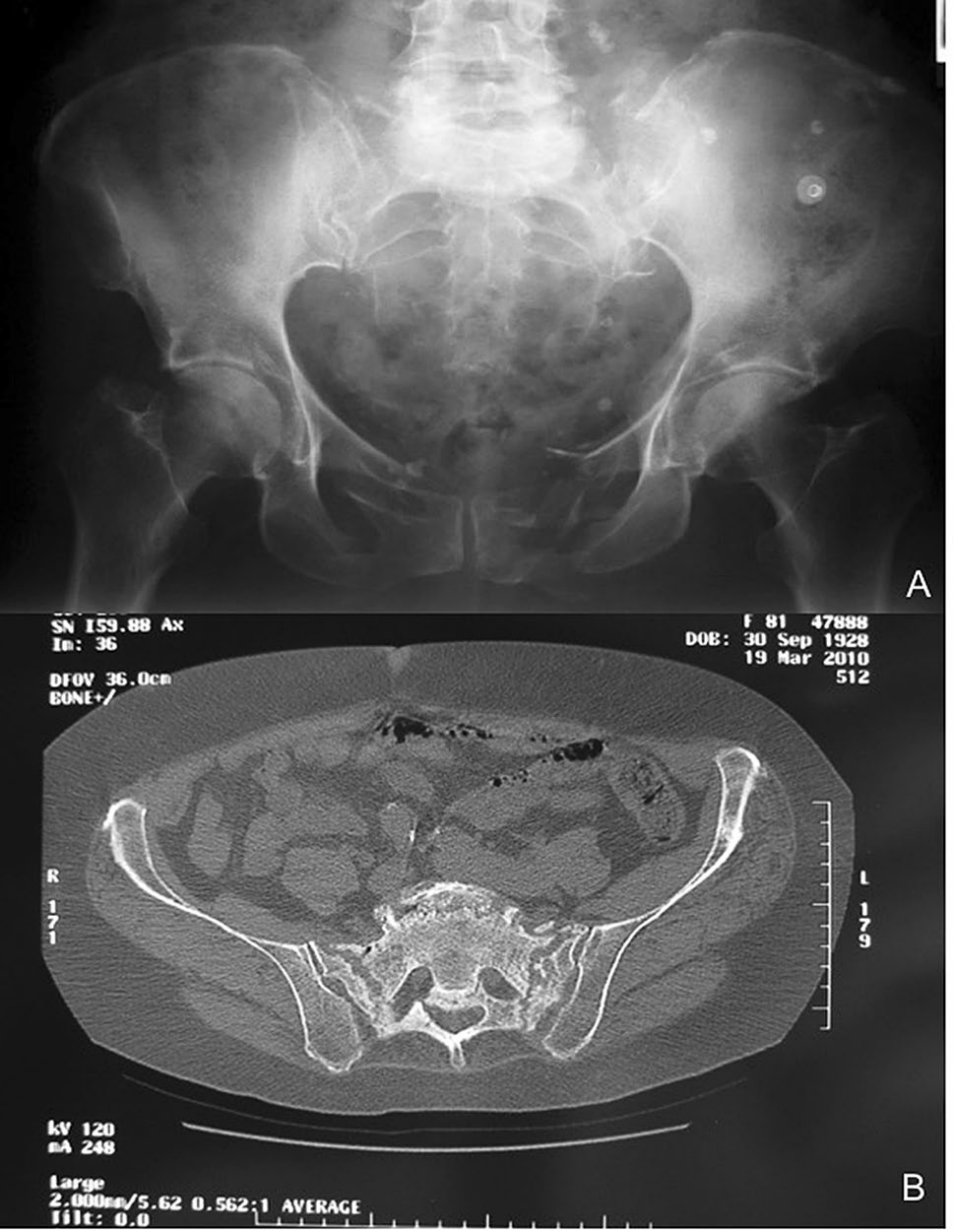
Preoperative X-ray and CT scan

All patients with trauma or persistent lower back pain received a standardized therapeutic procedure. Clinical data such as medical history, clinical examination, radiographs of the pelvis anterior–posterior, followed by computed tomography (CT scan, Fig. 1) of the pelvic in case of anterior pelvic ring disruption were performed. In cases of persistent lower back pain without apparent radiological findings the indication for a MRI scan was given.

All patients were initially mobilized with physical therapy under pain medication. Operation was proposed in fractures we considered as being unstable such as FFP 3 and FFP 4 and in some cases of FFP 2 fractures, whenever conservative therapy was unsuccessful and patients could not sufficiently be mobilized. Some patients initially refused surgery and were ultimately operated due to persistent immobilizing pain after a mean of 6.7 hospital days. We performed surgery at the earliest 3 days and at the latest 720 days after trauma (mean 55.5 days). Some patients had a long ambulatory history prior to our examination as the origin of the pain was not immediately detected. We used a MIPLCP as standard operative procedure for these indications even in FFP 2 fractures. Postoperatively patients underwent conventional X-ray controls.

Besides from the clinical aspect our criteria for surgery were radiological signs of instability and fracture progression. The FFP classification for fragility fractures of the pelvis was used for classification [12]. The classification was performed by a senior surgeon and revised by a highly experienced senior surgeon.

### Surgical procedure

**Fig. 2:**
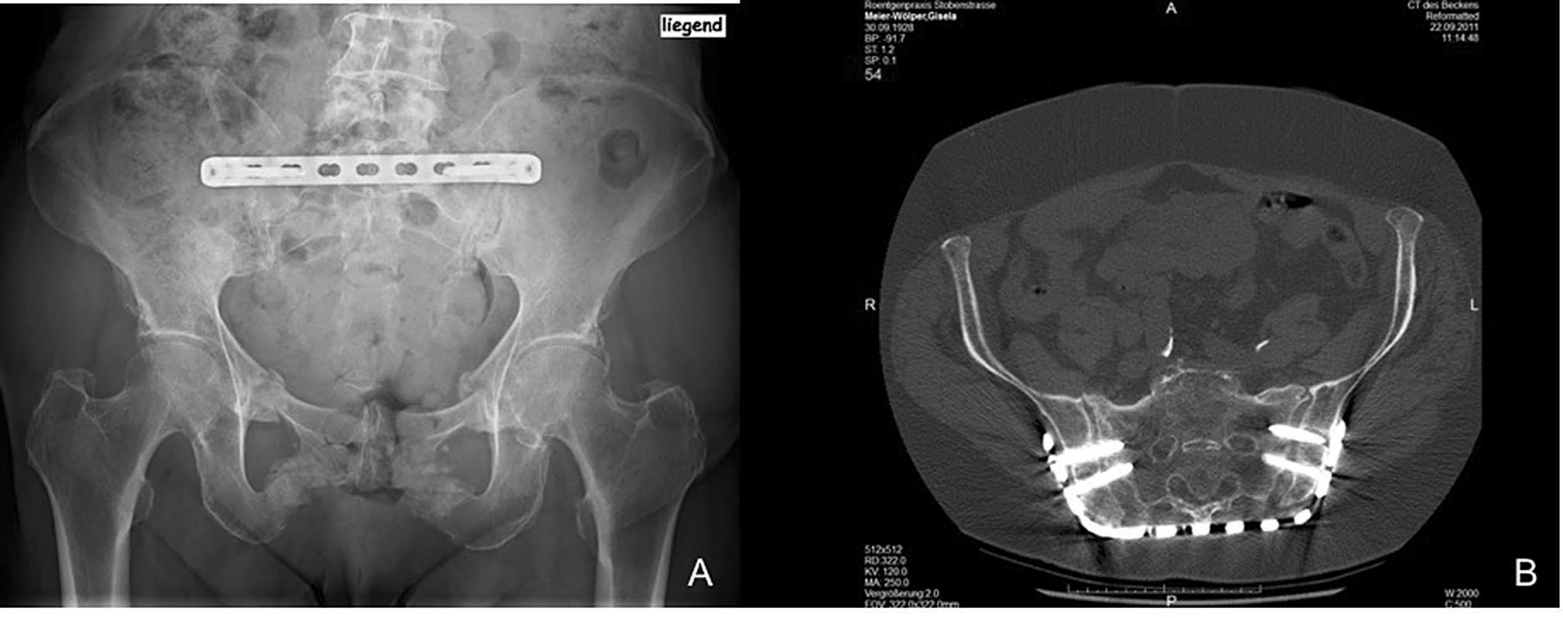
Follow-up X-ray and CT scan

The surgical procedure has been described earlier [21, 26]. In summary it was performed minimally invasive in prone position using a 4.5 locked compression plate (LCP) (manually bent) as an angular stable implant, placing angular-stable screws through the ilium into the massa lateralis of the sacrum (Fig. 2). Patients were mobilized according to their capacity with full weight bearing with or without crutches.

### Outcome parameters

We analyzed the demographic data of the patient cohort, including age, sex, living situation and care level. Furthermore we recorded Body Mass Index (BMI), American Society of Anesthesiologists (ASA) classification, time until surgery (in and out of hospital) and length of hospital stay, as well as perioperative complications. We analyzed whether patients went back to their former living situation after discharge or were institutionalized.

Preoperative diagnostics (CT, MRI) or where applicable scintigraphy were evaluated. Furthermore, we monitored the intraoperative radiation time and the operation time. The osteoporotic medication prior and after surgery was assessed and the follow-up period was monitored.

In order to assess the postoperative mobility and pain level we compared the Barthel index (activities of daily living) before discharge and in the follow-up, as well as the Iowa Pelvic Score and the Visual Analog scale (VAS). The fracture classification was done by a senior surgeon using the FFP classification.

We analyzed statistical data of the whole cohort concerning living situation pre and post trauma, hospital-stay, time till surgery, BMI, ASA, VAS, operation time, radiation dose, anti-osteoporotic medication and complications. In the follow-up we assessed VAS, living situation and mobility score (Iowa Pelvic Score) as well as anti-osteoporotic medication and whenever available x-ray control.

### Statistics

Statistic evaluation was performed with a Software R Version 3.4.0; R core team 2018.

## Results

### Demographics

**Fig. 3:**
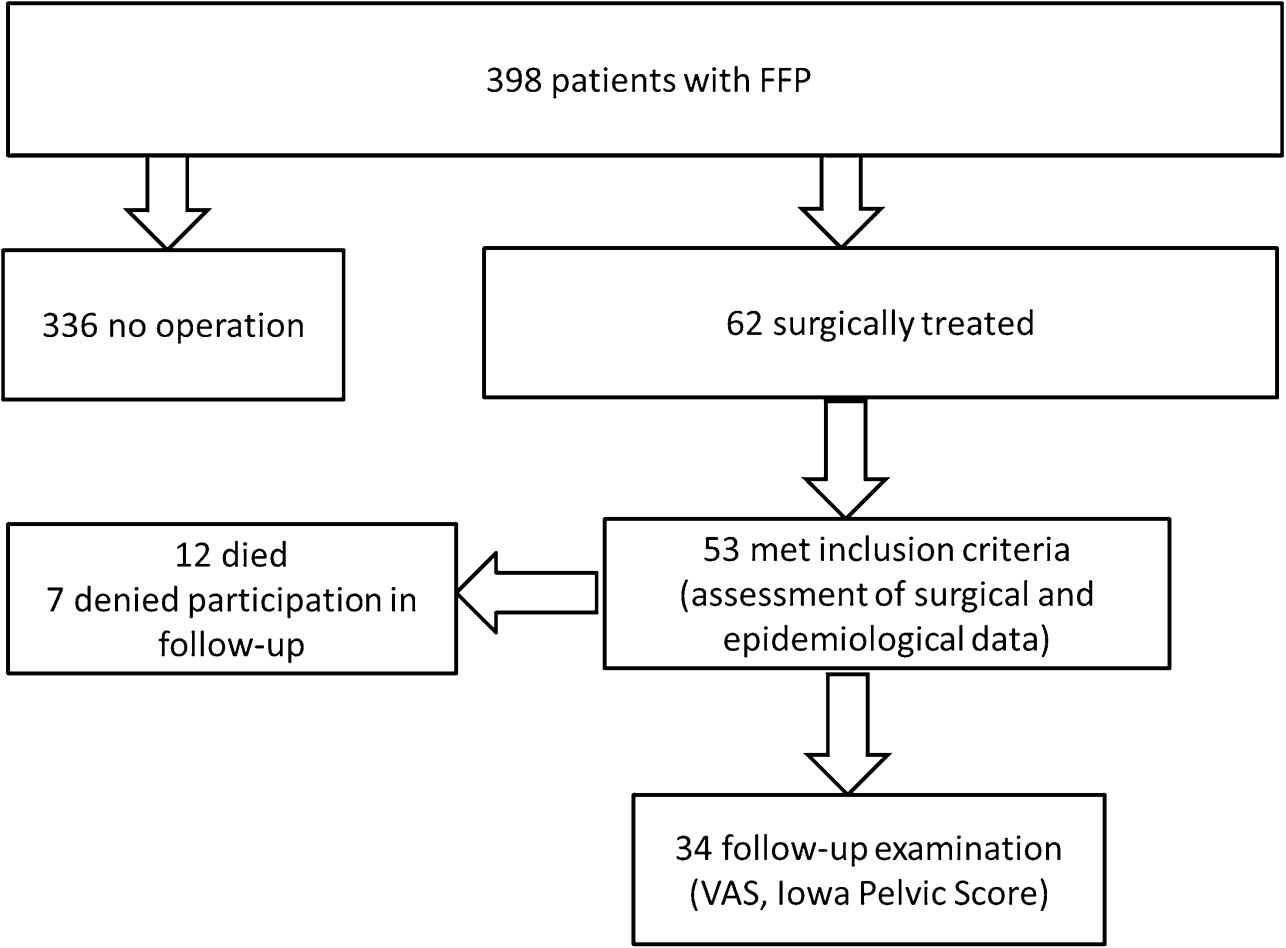
Patient cohort

Patient cohort (Fig. 3) Patient gender was 48 female versus five male. 34 out of 53 patients participated in the follow-up examination after written consent. 12 patients passed away in the meantime, seven denied to participate. Mean age was 79.1 (SD 7.8), with a BMI of 24.8 (SD 4.5) and ASA of 2.7 (SD 0.6).

In our present study we retrospectively analyzed data from 53 patients which were treated surgically with MIPLCP in one single unit in the time from 5/2007 until 6/2015. Follow-up examination was done from 2015 until 2018 by visiting the patients.

### Radiographic results and classification

FFP classification (performed by a senior surgeon) in our surgically treated collective was as follows: One FFP II a (2%), Three FFP II b (6%), Nine FFP II c (19%), One FFP III b (2%), 21 FFP III c (40%) and 18 FFP IV b (31%). In 25 out of 34 patients (32 out of 53) we have a follow-up x-ray examination. In these patients we did not see any implant loosening, all fractures were united.

### Surgical data

Mean radiation time was 9.42 s (SD 9.6) with a dose-area product of 70.1 mGycm (SD 58). Operation time was 52.3 min (31–117 min).

Postoperative surgery-related complications occurred in seven patients (13%). Notably one mal-positioning of a screw, one irritation of the sacral root, three wound infections, which needed revision surgery and two prolonged wound secretions which ceased without surgical intervention. 46 patients (87%) did not show any surgery related complication. In 36 patients we used an 11-hole, in ten patients a 10-hole, in six patients a 12-hole and in one patient a 9-hole 4.5 LCP (Synthes). The plate length was intraoperatively assessed depending on the width of the sacrum and the dorsal pelvis.

### Hospital stay

Mean hospital stay after surgery was rather long with 14.3 days (SD 4.9) as we tried to dismiss patients only after sufficient mobilization. Overall hospital stay was 21.2 days (10–43 days). All patients with fractures we considered as being stable underwent a trial of conservative treatment. In patients with FFP 3 or 4 we proposed surgical therapy, even though most patients initially denied surgery and were operated after failure of mobilization due to persistent pain. Mean time till surgery after admission was 6.7 days (SD 5.1) and ambulatory period before treatment 55.5 days (3–720 days).

In nine out of 53 (17%) patients we saw non-surgery related complications. Eight urinal tract infections occurred during the hospital stay or the rehabilitation period, one patient suffered from pneumonia (1.8%) and died during the hospital stay.

34 patients went to a rehabilitation clinic, whereas 18 did not. Mean pain level at admission was 4.8 (SD 2), pain level prior surgery was 4.3 (SD 2.6). Pain level at discharge was 1.97 (SD 0.9).

### Follow-up data

Prior to trauma four patients (7.5%) were living in a nursing home, two patients (4%) in an assisted living situation and three patients (5.5%) were in a short-time-care unit, whereas 44 patients (83%) were self-dependent. After surgery 42 patients returned to their former living situation. Five patients changed their living situation and moved to a nursing home, one patient, who formerly lived in a nursing home, died after surgery (pneumonia). Three patients moved to an assisted living situation and two patients, who were living in a short-time-care unit due to immobilizing pain prior to surgery, returned to an independent living situation. Overall 39 out of 53 patients were able to live independently after surgery, five out of 53 patients only needed little help (assisted living). Five patients were newly admitted to nursing homes after discharge.

Preoperatively, ten patients had a care level, compared to 19 patients after discharge, as in some patients the hospital stay uncovered preoperatively existing nursing deficits. In others the fracture let to the need of professional nursing care.

A subgroup of 34 patients (64%) were clinically examined at an average of 31.5 (6–90) month after surgery. The results of the subgroup showed, that pain level at post-hospital examination was 2.4 (scale 0–10) with an IOWA Pelvic Score (daily live activity assessment) of 85.6 (SD 11.1). Postoperative Barthel score (daily living activity) was 69.4 (SD 18.8) at discharge with a follow-up score of 95.1 (SD 8.4). 26 (74%) patients did not need any analgetic therapy at the follow-up examination. All patients were mobile.

Besides we evaluated osteoporotic medication prior and after surgery. Three patients (8.8%) were under osteoporotic medication before trauma and 17 patients (55%) at the follow-up examination.

## Discussion

Until now there is no golden standard surgical procedure for osteoporotic pelvis fractures and appropriate studies are rare or pending. The aim of our study was to investigate whether MIPLCP shows sufficient results compared to other methods.

The most commonly used method is the ilio-sacral screw fixation (with or without cement augmentation), sometimes with additional ventral plating, retrograde transpubic screw fixation or external fixation. Thus we compared our data to studies regarding percutaneous ilio-sacral screw fixation in patients with fragility fractures of the pelvis.

During our study period 398 patients with fragility fractures of the pelvis were identified, out of which 15% (*n* = 61) had to be surgically treated. 53 patients met the inclusion criteria and 34 were available for follow-up examination. We only retrospectively classified the surgically treated patients according to the FFP classification at that time. From 2016 onwards all patients at our hospital were thus classified. Results of ongoing studies are being evaluated.

Similar to other studies we found a mean age of about 79 (60–99) years with a predominance of women, which constituted 90% of our collective [16, 30–36].

In our data the mean hospital stay until surgery was 6.7 days (SD 5.1) and the mean hospital stay overall was 21.2 days (SD 7.7), which goes in line with other studies, where the mean hospital stay varies from 13 to 23.7 days [16, 31, 33, 34, 36].

Hopf et al. [16] describe in their collective a mean hospital stay until surgery of 9.2 (1–24) days, which is similar to our findings.

In the after-care patients were mobilized according to their capacity with full weight bearing to avoid additional risk factors and further decrease of bone quality and muscle mass.

In our collective mean time from symptom onset until surgery was 55.5 days (3–720). This is in accordance with the data by Eckardt et al. and Sanders et al. which detected a mean time from first symptom to operation of 33.1 (7–103) to 68 days, with mentionable 10% of the patients suffering more than 104 days [30, 35].

Analysing the FFP classifications that led to the surgical procedure we found data similar to Noser et al., whereas the patient collective of Eckhardt et al. consisted more from FFP 4 than FFP 3 fractures [30, 31].

In our own proceed patients were operated whenever mobilization with full weight bearing was not possible or when patients presented with increase of pain and dismobility after initial conservative treatment. Whenever suitable (no soft tissue problems, no immobility prior to the acute trauma), we used the MIPLCP as surgical procedure. In cases of vertical instability or severe dislocation of the ventral pelvic ring we performed a combination of ventral and dorsal fixation techniques, such as additional ventral plating or retrograde transpubic screw osteosynthesis. Additional ventral fixation let to exclusion of the study.

In our collective we recorded a mean x-ray time of 9.42 s (SD 9.66), a mean dose-length-product of 70.125 mGycm (SD 57.9) and an operation time of 52.3 (34–117) minutes (SD 13.9). The notable outlier of 117 min of surgical time for the MIPLCP is caused by an additional total hip replacement due to a severe arthrosis of the hip, which was performed at the same time. We found five studies regarding fragility fractures of the pelvis which assessed operation times. Sanders et al. recorded operation times of 26.6 min, Höch et al. of 27 min per screw with 33 of the 34 patients needing additional ventral fixation, the total operation time was not assessed. Eckhardt et al. recorded operation times of 79 min, König et al. of 45.25 min, Wong et al. described mean operation times of 93.7 min (SD 32.4) and 133 min (SD 38.3), depending on whether the procedure was planned in advance or intra-operatively [30, 32, 34, 35, 37]. Percutaneous screw insertion is typically associated with considerable fluoroscopy use, although there have only been a few studies that fully assessed radiation doses in FFP. Eckhardt et al. recorded an mean dose-length-product of 449.6 mGycm, Sanders et al. assessed a radiation time of 2 min (range 101–143 s) [30, 35].

Far more data is available concerning ilio-sacral screw osteosynthesis in polytraumatized patients. Radiation doses up to two minutes and a dose-length-product (DLP) of up to 952.4 mGycm are described in studies that examined polytraumatized patients treated with ilio-sacral screw osteosynthesis [38–41]. Fischer et al. registered operation times with an average of 84 min (SD 35.2) and a mean radiation dose of 336.7 mGycm (53–2238 mGycm) [38, 42, 43]. Osterhoff et al. registered operation times of 16 min (SD 7) per screw, radiation doses or times have not been assessed. Time to full weight bearing was 9 weeks (SD 4) in their collective [23]. Compared to these studies, our collective had low radiation doses and a moderate operation time. An analysis from the Tennessee Surgical Quality Collaborative suggests that duration of operation correlates with complications and higher risks, thus a fast and effective surgical procedure could add to the safety of this rather frail patient cohort [44]. Our data show, that the MIPLCP is a suitable technique even in patients with FFP TYP IV b fractures [42, 44, 45]. The technique of the dorsal plating is simple whereas the iliosacral screw osteosynthesis can sometimes be silent, especially if it is difficult to reach a good view with the c-arm. Our locking plate has a high stability, as it provides threedimensional fixation with four angularstable screws that are placed through a 4.5 Locking Plate into the massa lateralis of the sacrum via the ilium.

Especially in cases of dysmorphic sacra where screw placement can be demanding and in bilateral injuries the MIPLCP might prove to be an excellent treatment option, due to relatively easy placement and high stability [42, 45, 46]. Biomechanical studies have shown that even non-locking plates show equal stablility to iliosacral screws. A biomechanical study by Albert et al. demonstrated a strong fixation of the pelvis by using a 4.5 recontruction plate, which was comparable to the fixation with a transsacral bar [47, 48]. Our technique provides even greater stability, as the fixation is angularstable and an arthrodesis of the iliosacral joint is performed using 45–55 mm 4.5 locking screws.

The positive effect of percutaneous approaches on perioperative complications has been described previously, therefore we are using a minimally invasive approach [41, 49, 50]. Similar to the therapy of osteoporotic fractures in other regions of the body, the use of angular stable implants has significant potential to result in higher stability and pull-out forces than conventional implants [51–54]. In our data, we did not see any complication regarding implant failure, even though we only managed to get hold of follow-up X-ray examinations in 32 out of 53 patients. Our results are promising, but further prospective, comparative studies with defined follow-up are necessary.

In our collective we registered 13% surgery related complications. As non-surgery-related complications, urinal tract infections occurred in 15%. We had one case of pneumonia (1.8%) in a 99 year old patient, who died during the hospital stay. Hopf et al. found a complication rate of 20% which is equal to the findings in our population [16]. In other studies complication rates from zero to 41% are described [16, 30, 32–36, 42, 55, 56] (Table 1).

**Table 1:** Complications in FFP

Study	Cohort	Complication rate, total	Death	Postop	Surgery rated	Migration
Collinge et al. [56]	24	8%				
Höch et al. [55]	50	18%	4%			
Sanders et al. [35]	11	0%				
König et al. [33]	20	20%		10%	5%	5%
Balling et al. [36]	26/26	11.5%/15%			11.5%/15%	2%
Höch et al. [34]	34	32%		18%	15%	
Eckhardt et al. [30]	50	22%			4%	18%
Hopf et al. [16]	30	23%		10%	10%	3%
Wong et al. [32]	17			35%	6%	41%

In the Hopf data pain level at discharge was 1.8 on average, with a significant reduction compared to pain level at admission. In our study we found similar pain levels at discharge of 1.9 in the whole study cohort and 2.1 in the sub-group (*n* = 34), that was followed-up, resulting in a significant reduction of pain level from 4.8 at admission. In the follow-up examination we found a pain level of 2.35. Other studies reported VAS at follow-up varying from 0.36 to 3.4 [16, 32–36, 56]. Comparison of pain level at discharge (1.98) and in the follow up (2.4) in our collective to the findings in the Hopf paper (1.73 at discharge) shows no statistically relevant differences. In both findings a relevant reduction of the pain level at admission to the pain level at discharge could be noted [16] (Tables 2, 3).

**Table 2:** Surgery time, radiation, complications (polytraumatized patients)

Study	Operation time	Radiation	Complications	Surgery related
Hilgert et al. [41]	55 min	1.6 min per screw		
Fischer et al. [38]	84 min	336.7 mGycm		
Osterhoff et al. [23]	16 min/screw			18%
Zwingmann et al. [5]			29.3%/26.3%	5.9%/8.8%
Tosounidis et al. [40]	58.9 min	1.83 min		
Pieske et al. [39]	44.3–88.6 min	953.4/779.1 mSv

**Table 3:** VAS-Score, Operation Time, Radiation in FFP

Study	Cohort	VAS preop	Discharge	5–6 weeks	Follow-up	Op time	Radiation
Collinge et al. [56]	24	7.9	3.4	3.2	2		
Höch et al. [55]	50				2.6		
Sanders et al. [35]	11	9.1	3.4		2.4	26.6	120 s
König et al. [33]	20	7.4	1.7	0.85		45.25	
Balling et al. [36]	26/26	8.8/9.0	3.5/3.6				
Höch et al. [34]	34	6.7	2.7		3.4	27	
Hopf et al. [16]	30	6	1.8				
Wong et al. [32]	17				0.36	93.7/133	
Eckhardt et al. [30]	50					79	449.6 mGycm

All patients in our collective were immediately mobilized with full weight bearing, resulting in an excellent mobility at discharge and at follow-up examination.

The follow-up of our collective showed a low number of patients who had to be institutionalized after trauma and a large number (74%) which returned to their former homes and living conditions. Noser et al. describe institutionalization rates of up to 75% [31].

For follow-up score we decided to apply the Iowa Pelvic Score (IPS). Until now there is no consistently validated outcome scoring system for pelvic fracture outcomes. In our collective the IPS at follow-up examination was 85.6 (SD 11.1) on a range from zero to 100 with 100 being the best possible result.

Limitations of the study are: retrospective analysis with small sample size, only a subgroup that has been followed up with pain and activity level. No comparison group. Follow-up range between 6 month and 7.5 years due to retrospective study design over a period of 8 years (5/2007-6/2015).

## Conclusion

The data of our retrospective clinical study demonstrates that using the minimally invasive posterior locked compression plate is a simple, safe, quick and reliable stabilization technique for patients with fragility fractures of the pelvis with lower radiation times and equal complication rates as ilio-sacral screw fixation.

## Data Availability

Data supporting the here reported results are stored and available on the server of our hospital according to the security guidelines of data storage.
